# The Unique Case of Lumbar Dural Metastasis From Cervical Carcinoma on Pembrolizumab

**DOI:** 10.7759/cureus.32559

**Published:** 2022-12-15

**Authors:** Riwaj Bhagat

**Affiliations:** 1 Neurology, Conemaugh Memorial Medical Center, Johnstown, USA

**Keywords:** immune checkpoint inhibitor, therapeutic plasmapheresis, intravenous immunoglobulins (ivig), dural metastasis, guillain barre syndrome (gbs)

## Abstract

Lumbar metastasis is a rare manifestation of cervical carcinoma which may present as lower extremities symptoms. Immune-checkpoint inhibitors like pembrolizumab, used for the treatment of various metastatic carcinomas, have been linked to Guillain-Barré Syndrome (GBS), which is characterized by ascending weakness and areflexia. A 52-year-old female with metastatic cervical cancer on pembrolizumab for two months presents with lower back pain, progressive bilateral lower extremities ascending weakness, and areflexia of affected limbs. Cerebrospinal fluid analysis showed albuminocytologic disassociation. Spine magnetic resonance imaging showed L4-L5 fracture associated with metastasis and contrast-enhancing adjacent dura and nerve roots. Despite the clinical presentation being consistent with lumbar metastasis, the possibility of masked pembrolizumab-induced GBS presented a therapeutic challenge. Plasmapheresis conferred no clinical improvement and later nerve conduction study was unrevealing for GBS. Subsequently, laminectomy of lumbar vertebrae improved the symptoms. The unique clinical scenario of lumbar dural metastasis from cervical cancer on pembrolizumab, a condition with a similar clinical presentation, is explored in this case.

## Introduction

Dural or pachymeningeal metastasis occurs either from direct extension of skull or vertebrae metastasis or by hematogenous spread of the primary tumor. The most common primary tumor to metastasize in dura is prostate, breast, lung, and stomach carcinomas [1]. Lumbar metastasis usually presents as lower back pain, radicular nerve pain, motor and sensory deficits of the lower limbs, and sphincter dysfunction [2]. Immune checkpoint inhibitors (ICI), such as pembrolizumab, are being utilized to treat metastatic carcinomas that have a 0.25% incidence of Guillain-Barré Syndrome (GBS) [3]. This case presents the unique clinical scenario of lumbar dural metastasis with concurrent use of pembrolizumab, posing a therapeutic challenge for possible concealed pembrolizumab-induced GBS.

## Case presentation

**Figure 1:**
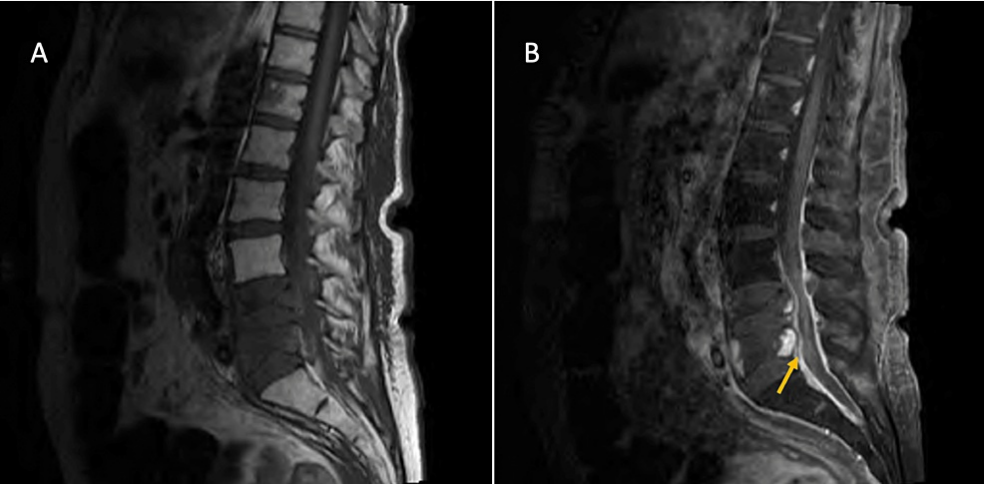
Magnetic resonance imaging of lumbar spine Lumbar magnetic resonance imaging (MRI) sagittal section, T1 sequence (A) without and (B) with gadolinium contrast, showing compression fracture at L4 and L5 vertebrae with contrast-enhancing mass and retropulsion and dural enhancement (arrow).

**Figure 2:**
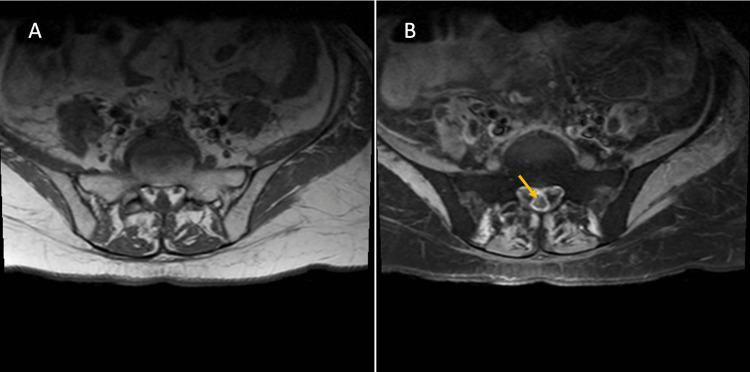
Magnetic resonance imaging of lumbar spine Lumbar magnetic resonance imaging (MRI) axial section, T1 sequence (A) without and (B) with gadolinium contrast, showing lumbosacral nerve root enhancement (arrow).

A 52-year-old female with a history of metastatic cervical cancer diagnosed 16 months ago and chemotherapy-induced peripheral neuropathy comes for the evaluation of progressive lower extremities weakness in the past two weeks and lower back radicular pain in the past two months. The lower extremities weakness was symmetric and started distally first and progressed in the ascending pattern affecting proximal muscles over the course of two weeks. The lower back pain radiated to both legs symmetrically. She had chronic numbness and tingling sensation on her feet extending to her knees. She did not have saddle anesthesia and fecal or urine incontinence. She had no preceding infection or vaccination. She was being treated with carboplatin and paclitaxel for the past 14 months and pembrolizumab for the past two months for cervical cancer. She has completed three doses of pembrolizumab 200 mg. On examination, she had bilateral proximal and distal lower extremity weakness with 0/5 Medical Research Council (MRC) strength. She had a chronic symmetrical loss of touch, vibration, and temperature sensation on her bilateral feet extending up to her knees and fingertips. Patellar and Achilles deep tendon reflexes were absent. Higher mental function, cranial nerve function, upper extremities strength, deep tendon reflexes, and coordination were intact. A blood workup showed thrombocytopenia, leukocytosis, anemia, and mildly elevated liver enzymes. Lumbar puncture showed normal opening pressure. Cerebrospinal fluid analysis (CSF) showed a white cell count of 3/cubic millimeter (cumm), red blood cells of 10/cumm, protein of 182 milligrams (mg)/deciliter (dl), and glucose of 78 mg/dl. Serum glucose was 110 mg/dl. CSF gram stain, culture, and cytology were negative. Magnetic resonance imaging (MRI) of the lumbar spine showed compression fractures and retropulsion at the fourth and fifth lumbar vertebrae with contrast-enhancing metastasis, dural enhancement, and L5 to S1 nerve root compression (Figure 1), suggesting osseous and dural metastasis and lumbosacral nerve root enhancement (Figure 2).

MRI of the cervical and thoracic spine and head with and without contrast was unremarkable. The presentation was consistent with lumbar metastasis and lumbosacral radiculopathy, however, the underlying masked pembrolizumab-induced GBS could not be ruled out without a nerve conduction study (NCS). Due to the lack of NCS in the inpatient hospital setting, a plan was established to treat both possible GBS and lumbar metastasis.

The patient was treated with five cycles of plasmapheresis for possible pembrolizumab-induced GBS which showed no clinical improvement in lower extremity strength. Intravenous immunoglobulin (IVIG) was not considered as a first choice therapy considering the potential loss of its therapeutic efficacy if plasmapheresis therapy is administered after IVIG. Back pain was managed by narcotics and gabapentin. The patient was also treated with physical and occupational therapy. A week after the completion of plasmapheresis, the patient underwent elective lumbar kyphoplasty. Two days after surgery, the patient started regaining lower extremities strength and by one week patient had 3/5 proximal and distal MRC strength in lower extremities. The sensation loss on the bilateral feet and fingertips persisted. The patient was then discharged to the rehabilitation center. A month after the initial hospital presentation, four limbs NCS was completed at the outside facility which showed no evidence of demyelinating polyradiculoneuropathy disease.

## Discussion

This case was challenging in terms of excluding underlying masked pembrolizumab-induced GBS and initial treatment selection. Usually, rapidly progressive ascending weakness of the bilateral legs with areflexia in the weaker limbs is a clinical marker of GBS. It proceeds to the peak clinical deficits in 2-4 weeks. Diagnosis of GBS is aided by the presence of albumincytologic dissociation in the CSF analysis. Prompt initiation of IVIG or plasmapheresis has proven to be beneficial for recovery [4]. Similarly, a short-term trial of steroids along with IVIG or plasmapheresis is considered the mainstay treatment for ICI-associated GBS [5].

Lumbar metastasis and GBS typically share similar clinical symptoms. To delineate the clinical presentation from lumbar dural metastasis a systemic review was conducted. The literature review was performed in PubMed including English literature till December 2022 with the search term “Lumbar,” “Dural,” “metastases” which yielded six case reports [6-11]. Cases with cervical and thoracic metastasis were excluded. Demographics, clinical presentation, examination, investigative findings, final diagnosis, and treatment were extracted and elaborated in Table 1.

**Table 1:** Review of cases with lumbar dural metastasis published till December 2022 Abbreviations: MRI: magnetic imaging resonance, CT: computed tomography, CSF: cerebrospinal fluid

Authors	Clinical presentation	Clinical examination	Investigations	Final diagnosis	Treatment
Vanderhoof et al. [6]	A 42-year-old male with vertebral chondrosarcoma comes with acute onset low back pain and urinary incontinence.	NA	MRI and CT myelogram showed multiple filling defects at L3-S1 consistent with intradural disease.	Metastatic chondrosarcoma	NA
Sikorski et al. [7]	An 11-year-old male with right Wilm's metastatic tumor comes with a fall, low back pain, and lower extremities weakness.	Bilateral 4/5 weakness in the gastrocnemius, tibialis anterior, and extensor hallucis muscles with diminished patellar and absent ankle reflexes bilaterally.	MRI showed intradural mass extending from L3 to S1 with gadolinium contrast enhancement.	Metastatic Wilm's tumor	Steroids, L3-L4 laminoplasty followed by midline durotomy.
Nagel et al. [8]	A 67-year-old male with carcinoid tumor of the thymus comes with left leg weakness for three weeks.	4/5 motor weakness in left hip flexors and knee extensors, decreased sensation in L4 dermatome.	MRI spine showed L5 epidural mass extending to L5-S1 foramen, epidural mass over T3 and bony fracture at T9.	Metastatic carcinoid tumor	Laminectomes and tumor resection.
Paterakis et al. [9]	A 31-year-old male comes with back pain, right leg progressive weakness, and sensory disturbances.	3/5 motor weakness and sensory loss in right leg.	5.2 mm X 1.3 mm L2-L3 intradural, extramedullary enhancing lesion with mass effect on cauda equina and similar lesion at L5.	Metastatic Ewing’s carcinoma	L1-L3 laminectomy and tumor resection followed by radio-chemotherapy.
Keddle et al. [10]	A 26-year-old male comes with progressive lower limb wasting, weakness, and sensory disturbances along with incontinence and bulbar symptoms.	Bilateral lower extremities asymmetrical distal more than proximal weakness, muscle wasting, absent patellar and ankle reflexes, reduced pinprick, and vibration sensation. Cranial nerves dysfunctions.	CSF analysis showed red blood cells >10,000/mm^3^, protein >6 gm/L, white blood cells 380/microliter, lymphocytes 377/microliter, glucose 3.7 gm/L, and 6 gm/L in serum, negative cytology. MRI Lumbar spine showed leptomeningeal nodular enhancing lesion at L3/L4. MRI brain showed enhancing nodular lesions on the 7^th^ and 8^th^ cranial nerve roots.	Choroid plexus papilloma with “drop metastasis” or leptomeningeal seeding	NA
Kim et al. [11]	A 43-year-old female with gastric adenocarcinoma comes with progressive lower back pain and radiating right leg pain over the period of 1 week.	No sensory or motor disturbances.	MRI showed enhancing Dural mass at lumbosacral junction with invasion to the right L5 and S1 nerve roots.	Metastatic gastric adenocarcinoma	Radiotherapy and later intrathecal methotrexate for recurring metastasis.

The ages of cases ranged from 11 to 67 years old and five of them were male. Majority of the cases presented with the sub-acute course (66.7%), lower back pain (66.7%), sensory disturbances (50%) on affected limbs, unilateral lower extremity weakness (33.3%), bilateral lower extremity weakness (33.3%), diminished or absent reflexes on affected limbs (33.3%), and incontinence (33.3%). On imaging, five cases had a lumbar intradural-extramedullary enhancing lesion and one case had a lumbar enhancing epidural lesion. One case has nerve root evasion [11], and one case had a mass effect on cauda-equina [9]. Only one case had CSF analysis which showed predominant red-blood cells, elevated corrected white blood cells, and protein in the setting of choroid plexus papilloma [10]. The final diagnosis was metastatic lesions from chondrosarcoma, Wilms’s tumor, choroid-plexus papilloma, carcinoid tumor, gastric adenocarcinoma, and Ewing’s carcinoma. The treatment approaches ranged from lumbar laminectomy, durotomy, tumor resection, steroids, chemotherapy, and radiotherapy specific to the type of primary tumor. None of the cases were treated with ICI. However, due to the lack of adequate CSF interpretation in the existing literature, the CSF profile from dural metastasis could not be determined. Albumincytologic dissociation can be seen in both spinal malignancies and GBS [12]. This review suggests the clinical manifestation from lumbar dural metastasis may resemble GBS, however, lower back pain is the key distinguishing feature between the two.

This is the first reported case of cervical carcinoma with lumbar dural metastasis. Consistent with existing literature, this case presented with lower back radicular pain, progressive bilateral lower extremities weakness, and absent reflexes on the affected limbs. Despite the presenting symptoms were consistent with lumbar metastasis, possible underlying pembrolizumab-induced GBS could not have been excluded without NCS, and thereby trial of plasmapheresis was performed, followed by laminectomy for dural metastasis.

## Conclusions

This is a unique case of lumbar metastasis from cervical carcinoma with concurrent use of ICI conferring a therapeutic challenge. According to the existing literature, lower back radicular pain is the key clinical feature of lumbar metastasis differentiating it from the GBS.
